# Methylation: a regulator of HIV-1 replication?

**DOI:** 10.1186/1742-4690-4-9

**Published:** 2007-02-02

**Authors:** Venkat RK Yedavalli, Kuan-Teh Jeang

**Affiliations:** 1 Molecular Virology Section, Laboratory of Molecular Microbiology, NIAID, the National Institutes of Health, Bethesda, Maryland, 20892 USA

## Abstract

Recent characterizations of methyl transferases as regulators of cellular processes have spurred investigations into how methylation events might influence the HIV-1 life cycle. Emerging evidence suggests that protein-methylation can positively and negatively regulate HIV-1 replication. How DNA- and RNA- methylation might impact HIV-1 is also discussed.

## Commentary

Biologically, methylation is executed by discrete enzymes (methyltransferases) which recognize DNA, RNA and proteins. Methylation of DNA, RNA or protein can regulate gene expression, RNA metabolism, and protein activity. Recently, protein methylation has stepped into the lime light due to the identification and characterization of protein arginine methyltransferases (PRMTs) which catalyze additions to arginine residues. PRMTs transfer one or two methyl groups from S-adenosylmethinine (SAM) to the guanidine-nitrogen atoms in arginine, forming methylarginine and/or S-adenosylhomocysteine [1]. PRMTs are ubiquitously expressed in cells; however, much is unknown about their regulated expression and stability [2].

Arginine methylation impacts cellular processes ranging from transcription, RNA processing, and cell signaling. PRMTs can function as transcriptional co-activators through remodeling chromatin and modifying histone tails. Methylation of histone H3 and H4 by CARM1 and PRMT1 is thought to contribute to a histone code [3,4] which programs gene expression. PRMTs also methylate non-histone proteins, such as CBP, to modulate co-activator function [5,6]. RNA binding proteins (RBPs) such as hnRNPs, Sam68, and Sm complex also have arginine-glycine repeats, a motif recognized and methylated by PRMTs. Methylation of RBPs may dictate activity as evidenced by findings that Sam68 and snRNPs require methyl addition to localize properly in the nucleus and assemble into appropriate multi-molecular complexes [7,8].

**Figure 1:**
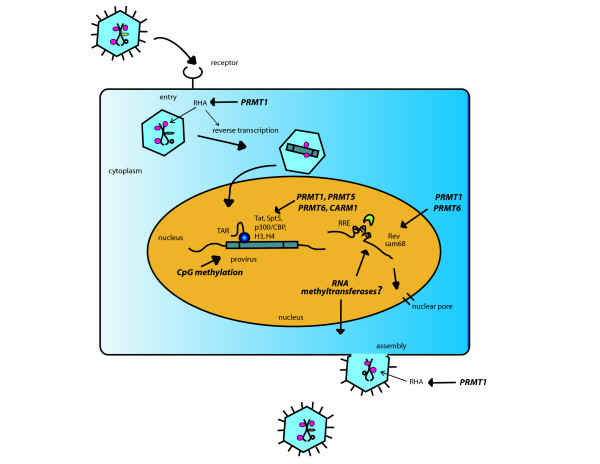
Schematic representation of steps at which methyltransferases may influence HIV-1 replication. Methyltransferases (*italics*) and their targeted steps in the HIV-1 life cycle are indicated.

Emerging evidence implicates methylation in regulated HIV-1 replication [9-12]. Now two new reports in *Retrovirology *[13,14] add to our understanding of how methylation could influence HIV-1 biology. In the first, Willemsen *et al*. used HIV-1 infected CEM T-cells and provirus transfected HEK293T cells to link protein methylation to virion infectivity. They employed adenosine periodate (AdOx), a general inhibitor of many methyltransferases, to address the effect of methylation on HIV-1 in tissue culture. Willemsen *et al*. found that AdOx treatment increased virus production from both adherent cells transfected with a proviral molecular clone and T-cells infected with HIV-1. Interestingly, the authors saw decreased p24 levels in infected CEM T-cells treated with AdOx, which was accompanied by substantially increased p24 amounts in the culture supernatants. This provocative observation suggests that methylation may regulate the processing and/or assembly of HIV-1 Gag protein. This interpretation makes sense because they also saw differences in the composition and size of virions produced in the presence or absence of AdOx, a finding consistent with perturbed virion assembly when methylation events are inhibited. Finally, AdOx treatment was found to significantly decrease HIV-1 infectivity; however, this decreased infectivity was overcome when HIV-1 virions were pseudotyped with VSV-G protein. The latter result invokes a methylation step in either HIV-1 envelope-receptor binding and/or virus envelope-uncoating in newly infected cells (Fig. 1).

In the second article, Invernizzi *et al*. described the novel methylation in HIV-1 Rev's arginine-rich motif by PRMT6. Here, they reported that PRMT6 binds Rev and decreases the stability of Rev in cells (Fig. 1). Additionally, methylated Rev bound the RRE (Rev responsive element in HIV-1 RNAs [15]) poorly and was associated with an attenuated Rev-RRE-dependent export of viral transcripts from the nucleus to cytoplasm. Thus, methylation in this context of HIV-1 infection provides a negative influence.

Collectively, the two new articles extend the emerging notion for methylation regulating HIV-1 biology. While the activity described by Invernizzi *et al*. stems clearly from PRMT6 methylation of Rev, the results accrued by Willemsen *et al*. paint a less clear mechanistic picture. Willemsen *et al*. used a broadly non-specific inhibitor of methylation, AdOx. AdOx is a competitive inhibitor of all SAM dependent methylation which includes protein-, RNA-, and DNA- modifications. Hence, the findings from Willemsen *et al*. while suggestive of an effect involving HIV-1 envelope cannot formally exclude contributions from Adox's action on unidentified RNA- or DNA- methylation.

So where do these reports lead us? The HIV-1 genome contains 9 open reading frames which can encode 15 proteins, and virus replication involves a complex interplay between viral and cellular proteins. What remains to be deciphered is the relative contribution of methylation on virus-factors versus cellular macromolecules. In this regard, Kwak *et al*. [12] have reported earlier that inhibition of arginine-methylation can also influence HIV-1 transcription. They showed that cellular transcription elongation protein SPT5 is a substrate of protein arginine methyltransferases PRMT1 and PRMT5, and that methylated SPT5 is less able to associate with RNA polymerase II and to assist Tat in efficient elongation of HIV-1 LTR-directed transcription.

Besides protein-methylation, there is compelling evidence that DNA-methylation influences HIV-1 replication. CpG methylation of LTR DNA has been reported to promote viral latency [16-19]. Some of these effects are explained by DNA-methylation impeding the binding of Sp1 and NF-κB transcription factors to the LTR promoter [20,21]. Indeed, activation of viral expression from latently infected cells does appear to correlate with loss of methylation of the HIV-1 LTR DNA.

Finally, what about RNA-methylation? RNA methyl modifications are involved in functional maturation of many species of cellular RNAs including mRNA, rRNA, tRNA, snRNA and snoRNA. Methylation of the RNA cap (7-methylguanosine cap) is a critical processing step for cellular and viral mRNAs. This action increases mRNA stability and promotes efficient export and translation. Other cap methyl modifications (e.g., hypermethylation – 2,2,7-trimethylguanosine modification of mRNA caps) have been identified in Oncorna viruses, Reo viruses, Sindbis virus, and *Caenorhabditis elegans *mRNAs [22-25]. The significance of these modifications remains undetermined. We have also recently identified a cellular RNA methyltransferase whose activity appears to upregulate HIV-1 gene expression (unpublished). Going forward, a better understanding of the varying complexities of methylation and their myriad effects on virus replication may unveil these processes as useful novel drug targets for HIV-1.

## Competing interests

The author(s) declare that they have no competing interests.
